# Protecting Privacy and Transforming COVID-19 Case Surveillance Datasets for Public Use

**DOI:** 10.1177/00333549211026817

**Published:** 2021-09

**Authors:** Brian Lee, Brandi Dupervil, Nicholas P. Deputy, Wil Duck, Stephen Soroka, Lyndsay Bottichio, Benjamin Silk, Jason Price, Patricia Sweeney, Jennifer Fuld, J. Todd Weber, Dan Pollock

**Affiliations:** 1 COVID-19 Response, Centers for Disease Control and Prevention, Atlanta, GA, USA; 2 Office of the Chief Operations Officer, Office of the Chief Information Officer, Centers for Disease Control and Prevention, Atlanta, GA, USA; 3 National Center for Birth Defects and Developmental Disabilities, Centers for Disease Control and Prevention, Atlanta, GA, USA; 4 US Public Health Service, Rockville, MD, USA; 5 Center for Surveillance, Epidemiology, and Laboratory Services, Centers for Disease Control and Prevention, Atlanta, GA, USA; 6 National Center for Emerging and Zoonotic Infectious Diseases, Centers for Disease Control and Prevention, Atlanta, GA, USA; 7 National Center for Immunization and Respiratory Diseases, Centers for Disease Control and Prevention, Atlanta, GA, USA; 8 National Center for HIV/AIDS, Viral Hepatitis, STD, and TB Prevention, Centers for Disease Control and Prevention, Atlanta, GA, USA; 9 Office of the Associate Director for Policy and Strategy, Centers for Disease Control and Prevention, Atlanta, GA, USA

**Keywords:** COVID-19, SARS-CoV-2, data privacy, de-identification, open data, data paper

## Abstract

**Objectives:**

Federal open-data initiatives that promote increased sharing of federally collected data are important for transparency, data quality, trust, and relationships with the public and state, tribal, local, and territorial partners. These initiatives advance understanding of health conditions and diseases by providing data to researchers, scientists, and policymakers for analysis, collaboration, and use outside the Centers for Disease Control and Prevention (CDC), particularly for emerging conditions such as COVID-19, for which data needs are constantly evolving. Since the beginning of the pandemic, CDC has collected person-level, de-identified data from jurisdictions and currently has more than 8 million records. We describe how CDC designed and produces 2 de-identified public datasets from these collected data.

**Methods:**

We included data elements based on usefulness, public request, and privacy implications; we suppressed some field values to reduce the risk of re-identification and exposure of confidential information. We created datasets and verified them for privacy and confidentiality by using data management platform analytic tools and R scripts.

**Results:**

Unrestricted data are available to the public through Data.CDC.gov, and restricted data, with additional fields, are available with a data-use agreement through a private repository on GitHub.com.

**Practice Implications:**

Enriched understanding of the available public data, the methods used to create these data, and the algorithms used to protect the privacy of de-identified people allow for improved data use. Automating data-generation procedures improves the volume and timeliness of sharing data.

Federal open-data initiatives that promote increased sharing of federally collected data^1-3^ are important for transparency, data quality, trust, and relationships with the public and with state, tribal, local, and territorial partners.^4^ These initiatives advance understanding of health conditions or diseases by making data available to researchers, scientists, and policymakers for analyses and other valuable uses. Data-sharing initiatives are particularly important during the COVID-19 pandemic, when data needs are constantly evolving and there is much to learn about the disease.

As part of a coordinated COVID-19 response, local jurisdictions share de-identified, patient-level data for each case with the Centers for Disease Control and Prevention (CDC). These data are sent daily in a combination of 3 formats—comma-separated values (CSV) files, direct data entry of case forms, and National Notifiable Diseases Surveillance System electronic case notifications—to CDC’s Data Collation and Integration for Public Health Event Response (DCIPHER) system. DCIPHER is a data management and analysis system that uses Foundry (Palantir Technologies) software, which allows analysis via R (R Core Team), Python (Python Software Foundation), and the analytic tool Contour (Palantir Technologies). The data are managed in the DCIPHER case surveillance pipeline, a series of linked programs that cleans, collates, de-duplicates, and transforms data to produce an analytical-ready epidemiologic dataset used by multiple CDC response teams. Data do not include direct identifiers, but they do include demographic characteristics, exposure history, disease severity indicators and outcomes, clinical data, laboratory diagnostic test results, and comorbidities (Figure 1).

**Figure 1:**
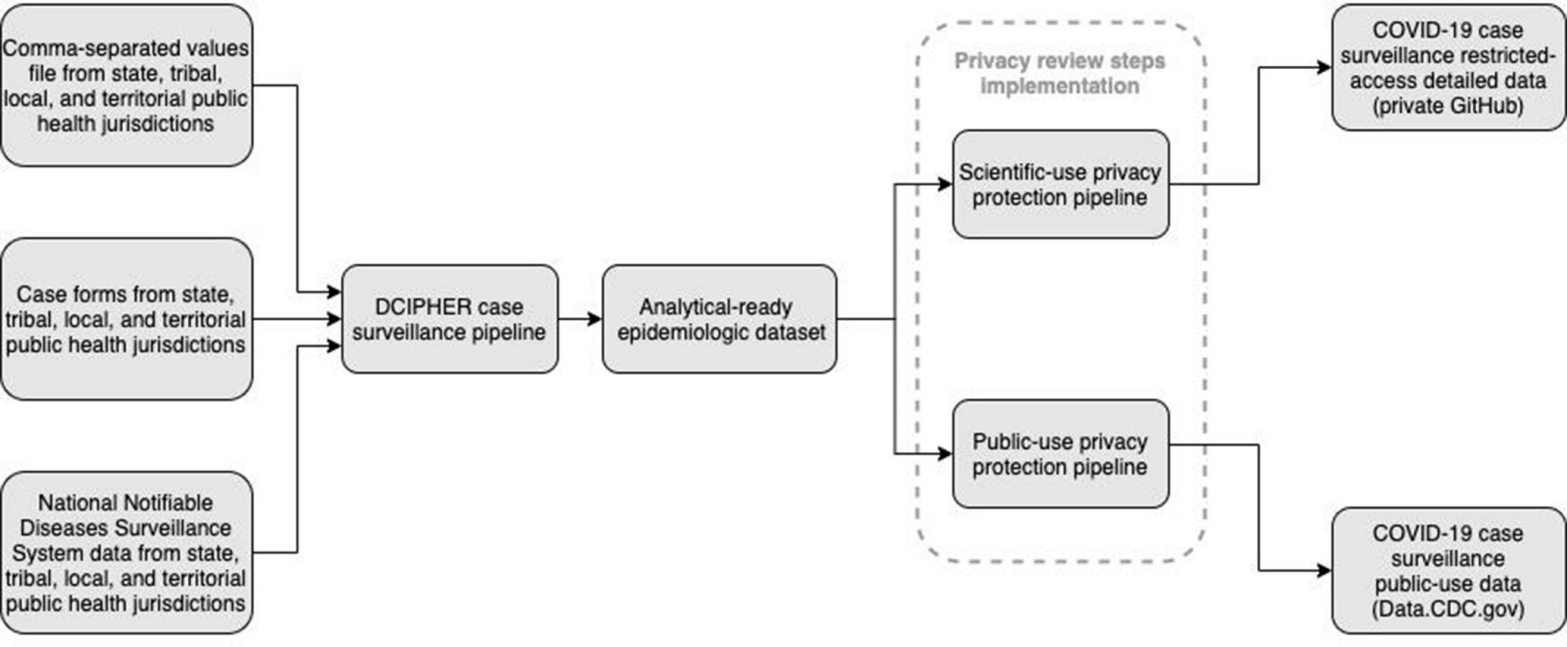
Creation of COVID-19 case surveillance public datasets from state, tribal, local, and territorial public health jurisdictions, Centers for Disease Control and Prevention, 2020. Abbreviation: DCIPHER, Data Collation and Integration for Public Health Event Response.

CDC’s Case Surveillance Section, the response group established to conduct surveillance activities and serve as the steward for case data, created a new process that transforms the epidemiologic dataset with privacy protection algorithms to systematically create de-identified subset data. This process contains automated workflows and R statistical software version 4.0.3 that implement and validate field-level suppression for *k*-anonymity^5^ and *l*-diversity^6^ levels to release microdata monthly in 2 de-identified public datasets. *K*-anonymity is a technique to reduce the risk of re-identification that can occur by linking datasets, and *l*-diversity is a technique to reduce the risk of revealing confidential information. First, COVID-19 Case Surveillance Public Use Data is a public-use dataset that has 11 data fields, is accessible via Data.CDC.gov,^7^ and has interactive visualization to allow the public to filter, sort, and perform exploratory analysis. Second, COVID-19 Case Surveillance Restricted Access Detailed Data is a scientific-use dataset that has 31 fields and more stringent privacy protections than the public-use dataset. It was designed to provide detailed information to scientists, statisticians, journalists, and researchers who sign a registration information and a data-use restriction agreement; access is through a private GitHub repository.^8^ GitHub is a third-party website that facilitates the downloading of data files as industry-standard, zip-compressed, CSV files.

To increase usability and foster transparency, this article describes dataset definitions, the design of the pipeline that created the datasets, and privacy protections.

## Materials and Methods

**Figure 2:**
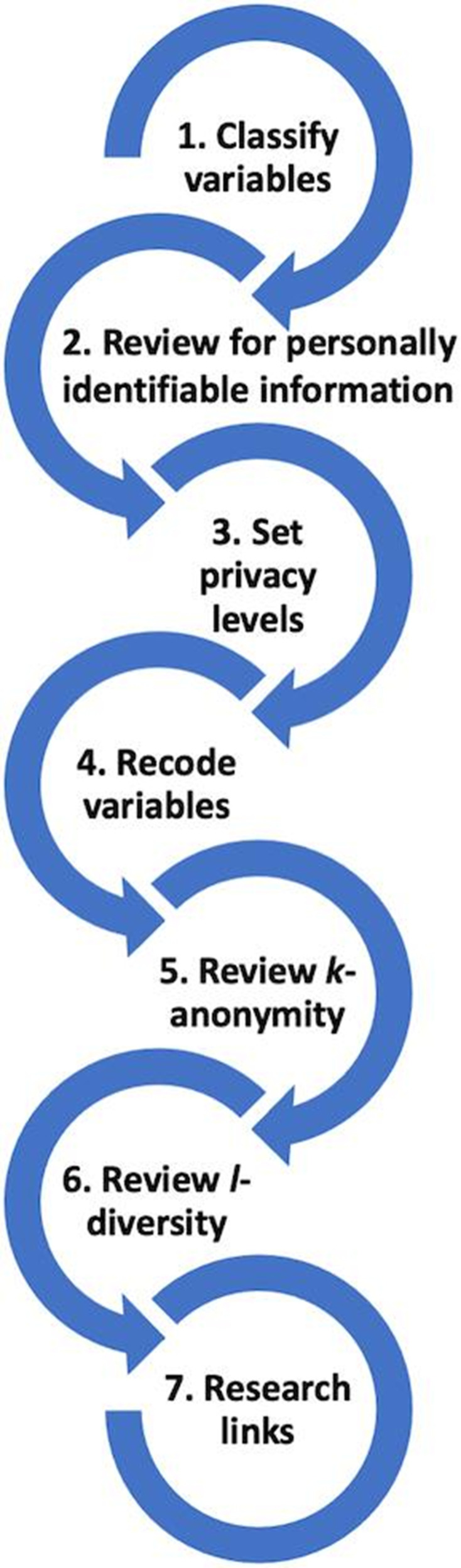
The 7-step process of privacy review implemented by the Centers for Disease Control and Prevention in the design of 2 public datasets for COVID-19 case surveillance in 2020.

Multiple groups in CDC’s emergency response organization worked together to design the 2 public datasets. The Surveillance Review and Response Group, a group established to improve data use for CDC’s COVID-19 response, worked with the Case Surveillance Section to use privacy heuristics, available guidance, and codes of practice^9,10^ to develop *Data Sharing Privacy Review Procedures*, a document that describes a 7-step process (Figure 2) that implemented requirements of CDC’s data release policy^11^ to protect privacy and publish useful and accessible data. We used this privacy review process to derive 2 datasets from the epidemiologic dataset (Figure 1). We created and verified datasets for privacy and confidentiality standards^12^ using Palantir Contour and R scripts and the sdcMicro package.^13^

Privacy procedures reduce the risk of re-identifying patients while providing useful information. To meet these privacy protection needs, not all variables can be released. Because the public-use dataset is widely accessible, these data are the most restricted. The scientific-use dataset is released only to approved researchers who sign a data-use agreement and includes more variables than the public-use dataset. Re-identification risk cannot be reduced to zero, but the systematic privacy review process is designed to make this risk low^14^ to protect people whose data contribute to these public datasets.

### Step 1: Classify Variables

We reviewed and classified all variables from the epidemiologic dataset according to their sensitivity into 1 of 4 categories: direct identifiers, quasi-identifiers, confidential attributes, and nonconfidential attributes. Direct identifiers are variables that would unambiguously identify a person (eg, name, address). Although CDC does not receive these types of data, we checked and confirmed each field to make sure that no identifying information appeared in an open-ended or free-text response. Quasi-identifiers are fields that may identify a person if they occur rarely enough in a dataset or could be combined with other fields or data (eg, age group, sex, county). Confidential attributes are sensitive information that would not commonly be known about a person (eg, date of first positive specimen [*pos_spec_date*]). Nonconfidential attributes are general information that cannot be used to identify a person but still may be combined with other data (eg, case status). We reviewed fields individually and as a combined set of fields in the dataset. From this review, we included, excluded, or transformed all potential fields to reduce sensitivity. For example, we excluded *date_of_birth*, so we created a generalized *age_group* field using 10-year bins with a top-coded bin for age ≥80.

We finalized the design of the datasets by identifying the fields included in the public-use and scientific-use datasets. We included fields based on analytical usefulness, public request, and privacy implications. We adjusted fields over time in response to feedback. For example, we added geographic fields for county to the scientific-use dataset and fields for race/ethnicity to the public-use dataset. We included geographic fields only in the scientific-use dataset.

As of December 4, 2020, the public-use dataset comprised 11 fields with 3 quasi-identifier fields (*sex*, *age_group*, *race_ethnicity_combined*) and 1 confidential attribute (*pos_spec_date*). As of December 4, 2020, the scientific-use dataset comprised 31 fields with 6 quasi-identifiers (*sex*, *age_group*, *race_ethnicity_combined*, *res_county*, *res_state*, *hc_work_yn* [health care worker status]) and 1 confidential attribute (*pos_spec_date*). These fields are used in subsequent steps to establish and check cell suppression levels (Table).

**Table:** Privacy characteristics used to create datasets and how they differ between the public-use and scientific-use datasets, developed in 2020 by the Centers for Disease Control and Prevention for design of 2 public datasets for COVID-19 case surveillance

Variable	Definition	Public-use dataset	Scientific-use dataset
No. of fields	Total fields	11	31
Privacy threshold	Minimum acceptable value for privacy calculations. *K* is the threshold for *k*-anonymity; *l* is the threshold for *l*-diversity.	*k* = 5 [minimum allowed records sharing quasi-identifier values] *l* = 2 [minimum allowed values of confidential fields by records sharing quasi-identifier values]	*k* = 5 [minimum allowed records sharing quasi-identifier values] *l* = 2 [minimum allowed values of confidential fields by records sharing quasi-identifier values]
Quasi-identifier fields	Dataset fields that may identify individuals	sex [sex] age_group [age group] race_ethnicity_combined [race and ethnicity]	sex [sex] age_group [age group] race_ethnicity_combined [race and ethnicity] res_county [county of residence] res_state [state of residence] hc_work_yn [health care worker status]
Confidential fields	Dataset fields that do not identify individuals but contain confidential information	pos_spec_dt [date of first positive specimen]	pos_spec_dt [date of first positive specimen]

### Step 2: Review for Personally Identifiable Information

We reviewed all data fields to confirm that they did not contain any personally identifiable information^11^ beyond what we classified in Step 1. We limited all data fields to categorical, date, and numeric values. We excluded all free-text data fields that potentially contained personally identifiable information.

### Step 3: Set Privacy Levels

We established privacy thresholds by defining the minimum acceptable size for the number of records in the dataset that share quasi-identifiers. Although no universal threshold is defined,^15^ a minimum level is suggested, with a common recommendation of 5 and uncommonly above 5.^16^ We set this level at 5 to be conservative and consistent with previous approaches used in public health.^17^ This means that no fewer than 5 records are allowed to share values from a single, or any combination of, quasi-identifier fields. We created workflows called “contour boards” in DCIPHER to automatically detect any combination of quasi-identifiers meeting our criteria for small cell suppression and set fields to the value “NA,” indicating that the original value was not applicable. We suppressed only field values; records remained in the dataset so researchers could identify when we applied suppression criteria. When suppressing fields, data managers made every effort to suppress as few fields as possible while meeting the privacy level (Table).

### Step 4: Recode Variables

We used common variable coding techniques in the case surveillance pipeline to clean and ensure uniformity of the responses in each field. We reclassified questions that were left unanswered in the case report form to “missing,” with the following exceptions: we recoded *age_group* to “unknown,” we recoded *res_state* to the reporting jurisdiction, and we left blank *res_county* and *county_fips_code* (county Federal Information Processing Standards [FIPS] code). We checked dates to detect illogical responses (dates in the future, dates before the COVID-19 pandemic) and set them to “null” until the jurisdiction updated the information. If reporting jurisdictions sent a blank value for the initial COVID-19 report date, we set the value to the date the data file was first submitted to CDC. The objective of the recoding process was to ensure consistency in applying suppression and to simplify the dataset for ease of use and analysis.

Case-based surveillance data are dynamic, and jurisdictions can modify and resubmit files when new information becomes available; therefore, records may change between releases. We include data only if the field for *cdc_report_dt* (CDC report date) is 14 days earlier than the day on which the datasets are generated. This practice allows data managers time to review responses and work with jurisdictions to correct data quality issues. The original release on May 18, 2020, used a 30-day window but was updated as improvements in data quality processes reduced the number of changes after 14 days.

### Step 5: Review *K*-Anonymity

Each time a dataset is generated, we review for *k*-anonymity. *K*-anonymity is a technique used to reduce the risk of re-identifying a person or linking person-specific data to other information based on a rare combination of quasi-identifiers. We use this technique to suppress quasi-identifier values so that each person in the released dataset cannot be distinguished from at least *k − 1* other people who share the same quasi-identifiers.^5^ This technique uses privacy thresholds established in Step 3 across all quasi-identifiers classified in Step 1.

**Figure 3:**
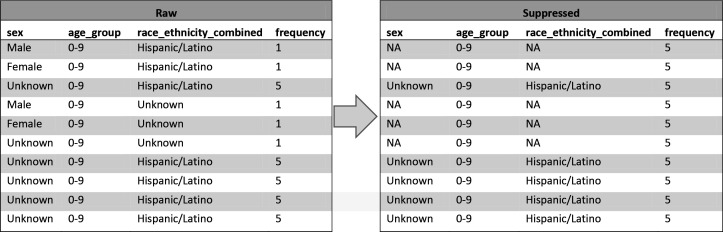
An example of how *k*-anonymity field suppression changes the values of quasi-identifier fields *sex, age_group*, *race_ethnicity_combined* to reduce the risk of re-identification of individuals in 2 public datasets developed by the Centers for Disease Control and Prevention in 2020 for COVID-19 case surveillance. When the frequency count of raw records with shared quasi-identifiers is below the *k* = 5 privacy threshold, suppressed data are produced with “NA” values for some quasi-identifiers so that the frequency increases to 5. Abbreviation: NA, not applicable.

To illustrate how *k*-anonymity is used to suppress quasi-identifier values and how it applies to the entirety of both datasets, we developed an example that uses only 10 records (Figure 3). Fields on the left are the raw data before suppression. The *frequency* field indicates the number of records in the dataset that have the same combination of quasi-identifiers. For example, for the first record, *frequency* is equal to 1, meaning that the combination of *sex*, *age_group*, and *race_ethnicity_combined* occurs only once in the data. Because we set *k* = 5 for these datasets and require 5-anonymity, we will suppress fields so that their quasi-identifiers occur at least 5 times. After suppression, *frequency* shows that each record’s quasi-identifiers occur 5 times. Note that records are never removed; in this example, because we suppressed the fewest fields possible to create a cell with 5 members, we suppressed only *sex* and *race_ethnicity_combined* and we were able to leave *age_group* unchanged. This example includes the 3 quasi-identifiers in the public-use dataset; an example for the scientific-use dataset using its 6 quasi-identifiers would function similarly.

After each time a dataset is regenerated through the privacy protection pipeline, data managers use R programs^12^ to verify that each generated dataset meets the levels established in Step 3. If the data managers detect any errors, they revise the pipeline to correct the bug and regenerate and retest the dataset until both processes are satisfied. At the end of this step, they verify each dataset to be 5-anonymous.

The number of times that fields are suppressed in each dataset varies with each monthly release and between datasets because suppression depends on the total number of rows in the dataset and on the number of included fields. Users should consider the amount of suppression within fields as they design and create analyses.

### Step 6: Review *L*-Diversity

As an extension of the *k*-anonymity check, Step 6 involves checking for *l*-diversity to reduce the risk of exposing confidential information for a person. *L*-diversity, another technique to protect confidential information, checks to ensure that for a group of people who share the same quasi-identifiers, at least *l* distinct values exist for each confidential attribute.^6^ These datasets require 2-diversity so that confidential variables cannot be determined in situations where records share the same quasi-identifier values.

**Figure 4:**
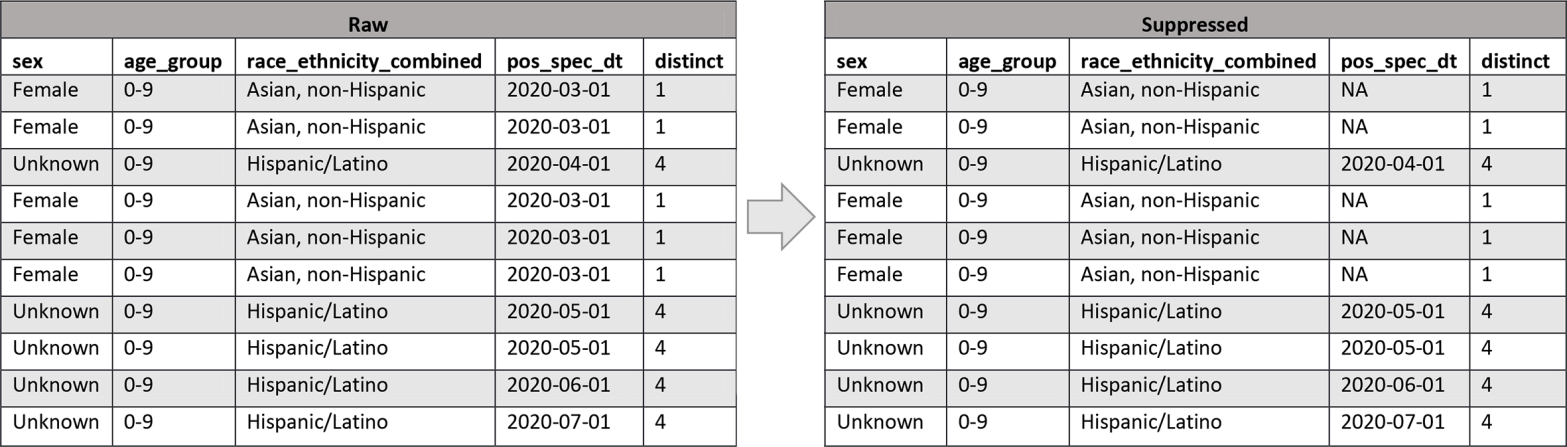
An example of how *l*-diversity field suppression changes values of the confidential *pos_spec_dt* field to reduce the risk of disclosure of personally identifiable information in 2 public datasets developed by the Centers for Disease Control and Prevention in 2020 for COVID-19 case surveillance. When the distinct count of raw records with shared quasi-identifiers *sex*, *age_group*, *race_ethnicity_combined* is below the *l* = 2 privacy threshold, suppressed data are produced with “NA” values for *pos_spec_dt*, preventing confidential information from being disclosed based on knowing a patient’s quasi-identifier values. Abbreviation: NA, not applicable.

We developed an example of how *l*-diversity is used to suppress confidential values in records to meet privacy levels (Figure 4). Again, the fields on the left are raw data. The *distinct* field indicates the number of unique *pos_spec_dt* confidential field values shared by all records with the same quasi-identifiers; notice that some records have a *distinct* of 1 because they all share the same *sex* field value of “female,” *age_group* field value of “0-9,” and *race_ethnicity_combined* field value “Asian, non-Hispanic,” and all share the same *pos_spec_dt* value of “2020-03-31.” Because our requirement for the dataset is 2-diversity, the confidential field is suppressed and set to “NA” to not reveal the *pos_spec_dt* value. The distinct value remains 1, but now the value is “NA” and cannot be known. This approach prevents someone knowing the date of the first positive specimen just because they know the person’s sex, age group, race, and ethnicity. Records are never removed; only field values are suppressed.

### Step 7: Research Links

Finally, in Step 7, to reduce the risk of the mosaic effect,^1^ we researched other publicly available datasets that could be linked by quasi-identifiers to identify people. The mosaic effect is a risk that is created when information in a dataset may not identify a person, but when the information is combined with information in other datasets, it might. This risk is decreased by using *k*-anonymity levels that reduce the number of rare combinations of quasi-identifiers that could be linked to other datasets; however, it is challenging to completely eliminate this risk. In May 2020, we reviewed the 13 COVID-19–related datasets published on Data.CDC.gov. We were not able to exhaustively search all available datasets, but we did review quasi-identifiers against the other 543 datasets published by CDC as of May 2020 with machine-readable metadata available through Data.CDC.gov. For the scientific-use dataset, researchers must confirm in the data-use agreement that they will not link to datasets, including CDC’s public datasets, to re-identify people.

## Results

CDC published the public-use dataset, containing 339 301 records and 9 fields, on May 18, 2020, at https://datacdcgov/Case-Surveillance/COVID-19-Case-Surveillance-Public-Use-Data/vbim-akqf. On May 29, we added the field *onset_dt* (date of symptom onset). On June 27, we added *race_ethnicity_combined*. As of December 4, 2020, the dataset contained 8 405 079 records, every case in the United States reported to CDC through November 19, 2020. To support the most users, CDC releases these data according to the FAIR Guiding Principles of findability, accessibility, interoperability, and reusability,^18^ including the use of machine-readable CSV formats and an open-standards–compliant application programming interface. As of December 4, 2020, the dataset was viewed more than 438 000 times and downloaded more than 24 000 times. An interactive visualization, https://data.cdc.gov/Case-Surveillance/COVID-19-Case-Surveillance-Public-Use-Data-Profile/xigx-wn5e, was used more than 15 000 times. As of December 7, 2020, Google Scholar (scholar.google.com) showed that 25 publications had referenced the public-use dataset. Source code is available at https://githubcom/cdcgov/covid_case_privacy_review.

CDC published the scientific-use dataset to a GitHub repository, containing 315 593 records and 29 fields, on May 18, 2020. On June 27, 2020, we combined *race* and *ethnicity* into *race_ethnicity_combined* and added *res_state*, *res_county*, and *county_fips_code*. As of December 4, 2020, the scientific-use dataset contained 8 405 079 records representing every case in the United States reported to CDC through November 19. Data dictionary, registration information, and instructions for the data-use restriction agreement are available at https://datacdcgov/Case-Surveillance/COVID-19-Case-Surveillance-Restricted-Access-Detai/mbd7-r32t. The dataset had been accessed by 94 researchers as of December 11, 2020, and Google Scholar shows 2 studies referencing these data.

## Discussion

Public datasets are needed for open government and transparency, promotion of research, and efficiency. Transparency of COVID-19 case data is important for fostering and maintaining trust and relationships with the public and state, tribal, local, and territorial public health partners.^19^ To balance the need to create and share public-use datasets with the protection of patients’ privacy and confidential information, we created a 7-step data-sharing privacy review.

Given the large number and variety of repositories for public datasets,^20^ as well as the large number of datasets contained in each repository, we were unable to develop a practical, systematic process for reviewing all public datasets and ensure with complete certainty that the risk of re-identifying patients in our datasets through the use of quasi-identifiers is completely eliminated. For example, a single popular repository for public research data, figshare.com, revealed 803 results for “COVID.”

We compensated for this limitation by reducing the number of variables, generalizing variables, and establishing conservative *k*-anonymity levels. As methods improve for comparing our data with other released datasets to rule out security concerns, we could include additional fields or apply more precise privacy levels, thus making our data more useful for analysis. This article describes work accomplished in 2020, but CDC efforts to release COVID-19 case surveillance datasets for public use continues, and we expect to release new public-use datasets using refined methods.

We developed the scientific and public-use datasets on independent timelines, and because we generated both datasets from the same common analytical sets, a linkability risk may exist. We accounted for this potential risk by carefully selecting variables for each dataset, using *k*-anonymity, and incorporating controls in the registration information and data-use restriction agreement that make this risk acceptable to CDC data stewards.

## Practice Implications

Systematic privacy review procedures are important for data engineering because they improve collaboration and iterative data design across systems, locations, and teams. Privacy review is complex, and requirements must be understood by epidemiologists, statisticians, data product owners, informaticians, analysts, health communicators, and data custodians so that they are implemented, tested, and applied reliably each time a dataset is updated. Automated computational privacy controls are important to meet the volume and schedule of data updates while reliably meeting privacy requirements; manual processes cannot fulfill these aims.

After we released these public datasets, we received user feedback that led us to make changes that improved data quality, such as consistently coding missing values, adding county coding, and accurately identifying state and county of residence. Public data are part of the data feedback loop throughout the data lifecycle where data users can identify and prioritize data features and fix bugs.

Through the creation of our 2 public datasets and implementation of computational privacy protections, CDC contributed to a knowledge base of COVID-19 data practices that will be used for design and publication of additional datasets beyond case surveillance. CDC had published 40 COVID-19 public datasets on Data.CDC.gov as of November 18, 2020. Currently, 2 datasets use these computational privacy protections; additional datasets will be published based on feedback and public health program priority.

CDC’s 2 public datasets are available to the public for review, for use in research, and to improve data transparency with partners. The practices and tools developed to design and release these data are available to other programs in CDC’s response to COVID-19 through the shared data privacy protection pipeline, privacy review procedures maintained by the Surveillance Review and Response Group, and computational privacy review software. With increased systematic releases of these public datasets and additional training and information available, we expect increased use and greater public health benefit.
